# Conditional survival of cancer patients: an Australian perspective

**DOI:** 10.1186/1471-2407-12-460

**Published:** 2012-10-08

**Authors:** Xue Qin Yu, Peter D Baade, Dianne L O’Connell

**Affiliations:** 1 Cancer Research Division, Cancer Council New South Wales, Sydney, Australia; 2 Sydney School of Public Health, The University of Sydney, Sydney, Australia; 3 Viertel Centre for Research in Cancer Control, Cancer Council Queensland, Brisbane, Australia; 4 School of Public Health, Queensland University of Technology, Brisbane, Australia; 5 School of Public Health and Community Medicine, University of NSW, Sydney, Australia; 6 School of Medicine and Public Health, University of Newcastle, Newcastle, Australia

**Keywords:** Conditional survival, Relative survival, Cancer registry, Australia

## Abstract

**Background:**

Estimated conditional survival for cancer patients diagnosed at different ages and disease stage provides important information for cancer patients and clinicians in planning follow-up, surveillance and ongoing management.

**Methods:**

Using population-based cancer registry data for New South Wales Australia, we estimated conditional 5-year relative survival for 11 major cancers diagnosed 1972–2006 by time since diagnosis and age and stage at diagnosis.

**Results:**

193,182 cases were included, with the most common cancers being prostate (39,851), female breast (36,585) and colorectal (35,455). Five-year relative survival tended to increase with increasing years already survived and improvement was greatest for cancers with poor prognosis at diagnosis (lung or pancreas) and for those with advanced stage or older age at diagnosis. After surviving 10 years, conditional 5-year survival was over 95% for 6 localised, 6 regional, 3 distant and 3 unknown stage cancers. For the remaining patient groups, conditional 5-year survival ranged from 74% (for distant stage bladder cancer) to 94% (for 4 cancers at different stages), indicating that they continue to have excess mortality 10–15 years after diagnosis.

**Conclusion:**

These data provide important information for cancer patients, based on age and stage at diagnosis, as they continue on their cancer journey. This information may also be used by clinicians as a tool to make more evidence-based decisions regarding follow-up, surveillance, or ongoing management according to patients' changing survival expectations over time.

## Background

Survival estimates for cancer patients are traditionally reported from the time of diagnosis such as five-year survival. It is useful for answering questions that many people ask about their prognosis when first diagnosed with cancer. For cancer patients who have already survived a number of years, survival expectations at diagnosis are too pessimistic because they include people who have already died. An ongoing question among these surviving patients is “now that I have survived for x years, what is the probability that I will survive another y years”. Over the past decade, the concept of conditional survival (CS) has emerged to directly address this question, because it provides cancer patients with survival expectations based on people who have reached a similar point in their cancer journey.

However, numerous previously published CS estimates have focused on one or a few cancer types, including cancer of the head and neck [1], stomach [2], colon [3-5], rectum [6], lung [7,8], breast [9] and melanoma of the skin [10-12]. Only a few published studies provided estimates for many cancer sites [13-18], and an even smaller number have included stratification by age group and stage at diagnosis [15-17]. Ellison et al. [14] acknowledged that a stratification of conditional survival estimates by age group at diagnosis provides more relevant clinical information for clinicians and cancer patients. Similarly other studies have acknowledged the limitation of excluding information about stage at diagnosis [13,15]. This has been shown to be an important prognostic factor for survival outcomes [19]. While it has been suggested that the impact of stage reduces and can disappear for long term conditional survival [16], there are currently no published Australian data describing conditional survival outcomes according to the stage at diagnosis.

This paper provides conditional survival estimates from New South Wales (NSW), Australia stratified by age group and stage at diagnosis for 11 major cancers.

## Methods

### Study population

New South Wales is the most populous state in Australia with a population of 7.2 million, approximately one-third of the Australian population. Age-standardised mortality rates from cancer in NSW are almost identical to the national rates (187.8 per 100,000 vs 187.1 per 100,00) [20]. The de-identified records of people diagnosed with one of 11 major cancers in NSW (Table 1) were obtained from the NSW Central Cancer Registry. The Registry maintains a record of all cases of cancer diagnosed in NSW residents since 1972, with notifications from multiple sources and linkage to death certificates. We included cases diagnosed in 1972–2006 and aged 15–89 years at diagnosis. Cases reported to the Registry through death certificate only or first identified at post-mortem were excluded.

**Table 1:** Conditional 5-year relative survival estimates, by type of cancer and number of years since diagnosis, for patients aged 15–89 years at diagnosis, NSW Australia 1998-2006

**Type of cancer**	**Number of patients**	**Estimated conditional 5-year relativesurvival (95% confidence intervals)**				
		**At diagnosis**	**1 year after diagnosis**	**3 years after diagnosis**	**5 years after diagnosis**	**10 years after diagnosis**
**Stomach**	5,193	28.5	51.3	79.4	91.0	95.0
		(27.2-29.8)	(48.8-53.8)	(76.2-82.6)	(87.6-94.3)	(91.0-98.9)
**Colorectum**	35,455	65.0	75.5	86.8	93.2	98.8
		(64.4-65.7)	(74.8-76.3)	(86.0-87.6)	(92.3-94.0)	(97.7-99.9)
**Pancreas**	5,213	5.6	21.9	64.3	81.8	94.7
		(5.0-6.3)	(19.2-24.7)	(56.9-71.7)	(73.8-89.9)	(86.1-103.4)
**Lung**	23,027	13.9	32.6	63.9	75.5	84.8
		(13.4-14.4)	(31.4-33.8)	(61.8-66.1)	(73.0-78.0)	(81.5-88.1)
**Melanoma**	27,888	92.6	93.8	95.9	97.3	99.0
		(92.1-93.1)	(93.2-94.3)	(95.3-96.4)	(96.7-97.9)	(98.4-99.6)
**Breast (females)**	36,585	88.6	88.8	90.4	91.6	93.3
		(88.2-89.0)	(88.3-89.2)	(89.8-90.9)	(91.0-92.1)	(92.6-94.1)
**Cervix**	2,368	73.2	81.4	90.7	95.1	95.3
		(71.4-75.0)	(79.3-83.5)	(89.0-92.4)	(93.7-96.6)	(93.9-96.8)
**Prostate**	39,851	90.2	90.7	91.2	90.0	89.6
		(89.6-90.7)	(90.0-91.3)	(90.4-92.0)	(89.1-90.9)	(87.5-91.6)
**Kidney**	6,940	64.0	78.1	87.2	89.6	88.8
		(62.7-65.4)	(76.4-79.9)	(85.5-89.0)	(87.7-91.4)	(86.1-91.4)
**Bladder**	6,154	62.5	75.1	86.0	89.8	91.5
		(61.0-64.0)	(73.2-77.1)	(83.9-88.0)	(87.7-92.0)	(89.1-94.0)
**Thyroid**	4,508	96.0	98.7	98.8	98.9	98.3
		(95.1-96.8)	(97.7-99.6)	(97.9-99.6)	(98.0-99.9)	(97.0-99.6)

The NSW Central Cancer Registry is the only population-based cancer registry in Australia that routinely collects information on spread of disease at diagnosis which had been used as an indicator of disease stage at diagnosis in this study. Medical coders from the Registry categorise stage based on information from statutory notification forms and pathology reports using a modified summary classification similar to the Surveillance, Epidemiology, and End Results (SEER) summary stage [21]. Categories are localised (stage I confined to tissue or organ of origin), regional (stage II spread to adjacent organs or tissues or stage III spread to regional lymph nodes), distant (stage IV with metastases to distant organs), or unknown stage (insufficient information available) [22].

Survival status was obtained through record linkage of the cancer cases in the Registry with the death records from the NSW Register of Births, Deaths, and Marriages and the National Death Index. All eligible cases were followed up to 31 December 2006 to determine survival status. This passive approach to follow-up may fail to ascertain all deaths and may incorrectly link some incidence and death records. A previous study investigating its completeness and accuracy found loss to follow-up to be uniform from 1980 to 1993 and estimated the resulting overestimation of relative survival to be a maximum of 2% [23].

This study was approved by the NSW Population and Health Service Research Ethics Committee (reference number: **2011/04/317**).

### Statistical methods

Estimation of relative survival overcomes the possibility that cause of death on death certificates may be inaccurate [24]. Relative survival is the ratio of the observed proportion surviving in a group of patients to the expected proportion that would have survived in an age- and sex-comparable group of people from the general population [25]. We calculated relative survival using the period approach [26], with cancer patients under observation between 1 January 1998 and 31 December 2006. In period analysis survival times can be left-truncated at the beginning of the period of interest in addition to being right-censored at its end. Expected survival was estimated using the Ederer and Heise (Ederer II) method [27]. Observed survival was measured from the month of diagnosis to the date of death or censoring (31 December 2006) whichever occurred first. Survival estimates were stratified by age group (15–49, 50–69 and 70–89) and stage at diagnosis separately. Stata 11 (College Station, TX: StataCorp) was used for all analyses together with publically available commands for estimating relative survival from Dickman et al. [28].

### Conditional survival

Conditional survival is defined as the probability of surviving an additional y years on the condition that the patient has survived x years. It is calculated by dividing the relative survival at (x + y) years after diagnosis by the relative survival at x years after diagnosis [8]. For each type of cancer, 5-year conditional survival is estimated at 1, 3, 5 and 10 years after diagnosis. We calculated the 95% confidence intervals assuming that CS follows a normal distribution and using Paul Dickman’s method for period analysis, the details of which can be found on his website [29].

## Results

A total of 193,182 cases were included in this study, with the most common cancers being prostate (39,851), female breast (36,585) and colorectum (35,455) (Table 1). Table 1 shows the 5-year relative survival estimates at diagnosis for each of the 11 selected cancer types, along with 5-year CS estimates for patients who have survived 1, 3, 5 and 10 years after diagnosis. Overall, 5-year relative survival tended to increase when conditional on increasing years after diagnosis and the greatest changes in CS occurred for cancers with poor prognosis at diagnosis for example, patients with aggressive cancers or those with advanced stage or at older age. For example, people diagnosed with lung cancer had an initial 5-year relative survival of 14%. However, their conditional 5-year relative survival increased to 33% after they survived one-year after diagnosis, and reached 85% if they survived 10 years after diagnosis. In contrast, 5-year relative survival was initially very high for men with prostate cancer (90%), with no change after surviving 10 years since diagnosis (90%).

Table 2 shows the 5-year relative survival estimates stratified by stage at diagnosis for each of the 11 selected cancers and conditional on having survived 1, 3, 5 and 10 years after diagnosis. The improvement in 5-year relative survival was greatest for cases with distant metastases when conditional on increasing years already survived whereas the impact on early stage cancers was much smaller.

**Table 2:** Conditional 5-year relative survival estimates, by type of cancer, disease stage and number of years since diagnosis, for patients aged 15–89 years at diagnosis, NSW Australia 1998-2006

**Type of cancer**	**Number of patients**	**Estimated conditional 5-year relativesurvival**				
		**At diagnosis**	**1 year after diagnosis**	**3 years after diagnosis**	**5 years after diagnosis**	**10 years after diagnosis**
**Localised**						
Stomach	1247	54.4	75.0	90.3	94.7	93.8
Colorectum	11450	89.6	93.3	95.1	97.4	99.0
Pancreas	849	9.9	24.7	65.1	76.5	98.5
Lung	5202	32.9	51.2	73.2	80.0	82.4
Melanoma†	20077	99.2	98.9	98.7	99.1	99.3
Breast (females)	19468	97.2	96.6	95.8	95.8	95.4
Cervix	1157	86.3	88.9	94.5	96.9	96.2
Prostate	18308	97.9	97.1	96.0	94.2	90.6
Kidney	3734	87.8	89.8	91.3	90.9	90.1
Bladder	3111	72.7	79.1	85.5	88.6	92.0
Thyroid	2920	99.7	100.0	99.5	99.4	99.2
**Regional**						
Stomach	1881	28.7	41.9	68.2	85.9	97.7
Colorectum	14965	67.2	71.5	82.0	90.1	98.5
Pancreas	965	8.2	19.3	61.3	84.2	95.9
Lung	4270	18.7	32.3	58.3	68.6	81.7
Melanoma†	4563	73.8	73.4	81.2	86.9	99.3
Breast (females)	12552	83.7	81.5	83.3	84.8	89.2
Cervix	616	59.7	67.5	80.8	89.0	92.4
Prostate	2237	87.8	88.3	89.0	88.3	81.7
Kidney	1202	54.7	65.3	81.3	89.4	82.8
Bladder	965	36.4	51.0	75.2	86.0	95.1
Thyroid	863	92.6	96.2	96.1	94.7	96.1
**Distant**						
Stomach	1289	5.2	20.6	81.2	93.4	95.5
Colorectum	5576	11.9	23.9	56.4	83.4	93.9
Pancreas	2059	2.3	20.4	62.4	83.2	98.7
Lung	8000	2.8	14.4	59.2	83.5	98.7
Melanoma†	1222	32.3	54.6	79.3	84.1	91.2
Breast (females)	1769	39.4	48.6	59.8	67.6	82.5
Cervix	149	18.4	40.0	71.2	76.3	93.6
Prostate	1577	16.8	25.9	43.4	53.6	80.7
Kidney	1031	6.4	20.3	59.1	65.9	76.7
Bladder	376	4.6	17.3	52.5	92.6	73.6
Thyroid	161	50.0	70.9	79.7	85.9	91.1
**Unknown**						
Stomach	776	27.7	53.9	81.7	88.8	85.3
Colorectum	3464	64.5	77.8	87.9	92.1	96.8
Pancreas	1340	6.9	23.3	67.7	81.3	77.5
Lung	5555	10.9	21.9	54.8	71.2	84.9
Melanoma†	2026	94.4	93.6	94.2	95.1	99.0
Breast (females)	2796	78.5	81.0	86.6	90.4	93.9
Cervix	446	74.9	83.4	90.6	95.7	92.2
Prostate	17729	90.0	88.7	88.6	86.9	89.4
Kidney	973	51.5	66.8	79.6	85.7	88.7
Bladder	1702	69.8	79.7	89.5	92.5	88.2
Thyroid	564	94.2	98.6	99.4	99.9	96.5

† Thickness of the lesion was also used to categorise disease stage at diagnosis for melanoma [T3 (thickness = 2.01-4.0 mm) and T4 (thickness > 4.0 mm) stages were grouped with ‘spread to regional lymph nodes’ as ”regional”]. Since thickness data prior to 1983 were considered unreliable, we used melanoma data from 1983 onward for the stage-specific survival estimates.

**Figure 1:**
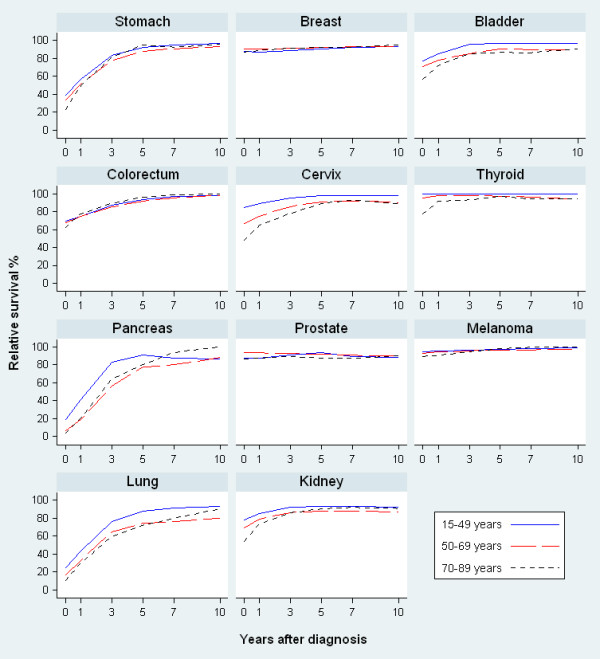
Age-specific conditional 5-year relative survival at 0, 1, 3, 5, 10 years after diagnosis, for patients aged 15–89 years at diagnosis, NSW Australia 1998–2006.

**Figure 2:**
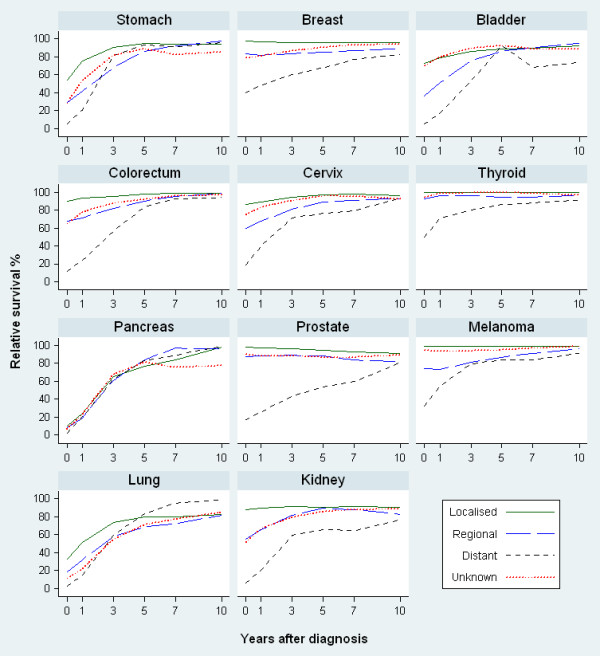
Stage-specific conditional 5-year relative survival at 0, 1, 3, 5, 10 years after diagnosis, for patients aged 15–89 years at diagnosis, NSW Australia 1998–2006.

Age-specific and stage-specific conditional 5-year relative survival at 0, 1, 3, 5 and 10 years after diagnosis for each selected cancer are also presented graphically in Figures 1 and 2. For most cancers, the age or stage differential in survival at diagnosis generally decreased over time except for cancers of the lung and pancreas.

## Discussion and conclusion

This study provides quantitative evidence that Australian cancer patients who are still alive ten years after their cancer diagnosis, even those diagnosed with advanced stage disease or at older age, have, at that moment, a much better survival outlook over the next five years than they did at diagnosis. The information is important for cancer patients as they face important life decisions in trying to plan their remaining life, and to provide evidence-based optimism as they continue living after their initial cancer diagnosis.

When 5-year relative survival exceeds 95%, the excess mortality is minimal, and so the survival for this group is considered similar to the general population with the same age structure [15,16], although this does not necessarily indicate cure of cancer. In NSW, we found that patients who were diagnosed with cancer of the colorectum, cervix and thyroid, and melanoma of the skin had similar mortality expectations after surviving 10 years since diagnosis. This was consistent with another Australian report [13]. Our results of 5-year CS after surviving one year since diagnosis were also consistent with a recent international comparison of cancer survival in which NSW data for colorectum, lung and female breast cancer were also included [18]. Their published CS rates for the three cancers in NSW [18] were slightly higher than those reported here as their estimates were age-adjusted, which would have the effect of increasing overall survival by reducing the weight given to the poorer survival among older people. The overall consistency of these results [13,18] with those we reported for overall CS during an almost identical study period provides indirect confirmation of our findings. The strength of our study is that we presented age- and stage-specific CS in addition to the overall CS. By presenting age- and stage-specific CS estimates for 11 major cancers in one geographically defined population, clinicians, cancer patients and their support networks can compare temporal and age-specific patterns in CS across multiple cancer types, thus gaining a greater understanding of the ongoing survival expectations faced by cancer patients.

Our results are consistent with many international studies including those in Europe [15,16] and North America [14,17] for a variety of individual cancer types. Our 5-year relative survival estimates conditional on surviving 5 years after diagnosis were very close to those for major cancers reported in Canada including cancer of the colorectum, lung, breast and melanoma [14]. The overall patterns of stage-specific CS for major cancer types from SEER data [17] and our data were also very similar particularly when accounting for any differences in age distribution: the increase in 5-year relative survival when conditional on more years already survived was greatest for later stage cancer and also the more fatal cancers but the stage differential in survival tended to reduce over time. Our stage-specific 5-year relative survival estimates conditional on having survived 5 years were very similar to those from SEER data [17] for localised and regional stage cancers of the colon and rectum, lung, breast and melanoma. However, considerable differences were observed for distant or unknown stages which may reflect different case mix in these two groups of patients due partly to more stringent criteria and data quality control being used in the SEER system than in Australia.

Regarding the effect of age at diagnosis on CS, patients diagnosed at older ages tended to have lower relative conditional survival, but this effect reduced substantially after surviving 5–10 years for most cancers. The overall pattern of age-specific CS estimates were similar to those from other studies in Europe [15,16] and US [17], although different age categories make it difficult to compare specific estimates. When we reanalysed our data with age categories matching those in a European study [15], we found that age-specific CS was consistently higher in NSW, apart from identical results for thyroid cancer for the three younger age groups (data not shown). The overall survival differences for three major cancers of the colorectum, lung and female breast between Australia and Europe had been confirmed by a recent study comparing survival from four major cancers between Australia, Canada and several European countries [18]. As suggested in that study [18], the possible explanations for these survival differences may be due to later diagnosis or differences in treatment in the European population.

As was noted in most previous studies, the greatest differences in conditional survival were for those cancers that had initially poor survival, such as lung or pancreatic cancer. This study confirmed a similar pattern for those cancers diagnosed at an advanced stage. While there was a substantial impact of disease stage on survival expectations at diagnosis, for most types of cancer this stage differential decreased as time since diagnosis increased, a pattern that has been reported in many international studies [2,8,15,17]. Unfortunately, because of the high initial mortality, the number of people who survive to enjoy this greater survival is low.

However the fact remains that of the people who do survive more than ten years after diagnosis, many continue to have poorer survival expectations than the general population with the same age structure. This could relate to the impact of the co-morbidities associated with the initial cancer diagnosis (for example while smoking causes lung cancer it is also associated with increased risk of cardiovascular diseases); the late recurrences of the primary cancer or secondary tumours; or the late side effects of treatment [15,16]. These ongoing reduced survival expectations for people diagnosed with late stage cancer in particular has substantial implications for health care providers in providing regular surveillance and monitoring even when the patients have survived at least ten or more years after the initial cancer diagnosis.

There are three widely used methods for estimating expected survival for relative survival analysis, commonly known as the Ederer I [30], Ederer II [27] and Hakulinen [31] methods. The Hakulinen method [31] was widely used in many international studies of cancer survival using population-based data including the most recent EUROCARE-4 study [32] and CONCORD study [33]. However, there is a growing consensus among researchers in relative survival analysis using population-based cancer registry data that the Ederer II method is more preferable [34-36], although relative survival estimates, using any of these methods, are generally very similar. Following this recommendation, we used the Ederer II [27] method in our estimation of relative survival. More recently, a modified Ederer II estimator has been proposed which is obtained by weighting the individual observations with their population survival [36]. The authors recommend the use of this new estimator when comparing cancer survival between countries because it is believed that this is the only unbiased estimator [36]. Another recent simulation study provided evidence that this estimator [36] is only unbiased for net survival when compared with other widely used estimators (including Ederer II [27] and Hakulinen [31] estimators) [37]. As the new estimator has not been used within the context of period analysis, future research in this area may be warranted.

Strengths of this study are the statewide population-based cancer registry data including information about the stage at diagnosis and multiple cancer types. This makes our study more representative and comprehensive than many other studies. The similarity between our estimates for all stages of cancer at diagnosis and those for another Australian state [13] provide optimism that the stage-specific CS estimates can be generalised nationally. We were unable to adjust for treatment, which may have impacted survival estimates through initial remission of cancer but may also cause longer-term adverse complications. Although mortality information was obtained by matching against the National Death Index, it remains possible that some deaths were missed, thus artificially inflating survival estimates. However since the matching process was not conditional on cancer type, it is unlikely that this would influence comparisons of conditional survival across cancer types.

In conclusion, these data provide important information for cancer patients, based on age and the stage at diagnosis, as they continue on their cancer journey. This information should be used by clinicians as a tool to make evidence-based decisions regarding follow-up, surveillance, or ongoing management according to their patient’s changing survival expectations over time.

## Abbreviations

CS: Conditional survival; NSW: New South Wales; SEER: Surveillance, Epidemiology, and End Results.

## Competing interests

The authors declare that they have no competing interests.

## Authors’ contributions

XQY obtained the data for the study, did the data analysis with assistance from PDB, drafted the Methods and Results sections; PDB drafted the Introduction and Discussion sections; DLO revised the drafts critically. All authors read and approved the final version of the manuscript.

## Pre-publication history

The pre-publication history for this paper can be accessed here:

http://www.biomedcentral.com/1471-2407/12/460/prepub
